# Topical ripasudil stimulates neuroprotection and axon regeneration in adult mice following optic nerve injury

**DOI:** 10.1038/s41598-020-72748-3

**Published:** 2020-09-24

**Authors:** Euido Nishijima, Kazuhiko Namekata, Atsuko Kimura, Xiaoli Guo, Chikako Harada, Takahiko Noro, Tadashi Nakano, Takayuki Harada

**Affiliations:** 1 Visual Research Project, Tokyo Metropolitan Institute of Medical Science, 2-1-6 Kamikitazawa, Setagaya-ku, Tokyo, 156-8506 Japan; 2 Department of Ophthalmology, The Jikei University School of Medicine, Tokyo, Japan

**Keywords:** Eye diseases, Trauma, Neurodegeneration, Experimental models of disease

## Abstract

Optic nerve injury induces optic nerve degeneration and retinal ganglion cell (RGC) death that lead to visual disturbance. In this study, we examined if topical ripasudil has therapeutic potential in adult mice after optic nerve crush (ONC). Topical ripasudil suppressed ONC-induced phosphorylation of p38 mitogen-activated protein kinase and ameliorated RGC death. In addition, topical ripasudil significantly suppressed the phosphorylation of collapsin response mediator protein 2 and cofilin, and promoted optic nerve regeneration. These results suggest that topical ripasudil promotes RGC protection and optic nerve regeneration by modulating multiple signaling pathways associated with neural cell death, microtubule assembly and actin polymerization.

## Introduction

Traumatic optic neuropathy is a common clinical problem that induces retinal ganglion cell (RGC) loss and optic nerve atrophy. Recent studies using an optic nerve crush (ONC) model in rodents have shown that neuroprotection and axon regeneration are promising strategies for the treatment of optic nerve injury^1–3^. One of the key molecular pathways that suppresses axon regeneration is the Rho/Rho-associated protein kinase (ROCK) signaling, which mediates the effects of myelin-associated axon growth inhibitors such as Nogo and repulsive guidance molecule^4^. Blocking Rho/ROCK signaling reversed the inhibitory effects of these molecules and stimulated axon regeneration and functional recovery in animal models of spinal cord injury^5^. In ONC models, intravitreal injections of two ROCK inhibitors, fasudil and Y-27632, showed different effects on optic nerve regeneration^6,7^. Y-27632 significantly promoted regeneration of rat and cat optic nerve fibers, whereas fasudil did not. These results suggest that efficacy of ROCK inhibitors in promoting axon regeneration is quite different among individual inhibitors.

Ripasudil is the first ophthalmic solution approved for the treatment of glaucoma and ocular hypertension in 2014, and its inhibitory effects are more potent than fasudil and Y-27632. Ripasudil lowers intraocular pressure (IOP) by altering the arrangement of cytoskeleton and focal adhesions in trabecular meshwork cells, which increases the outflow of the aqueous humor^8,9^. We previously reported that topical ripasudil prevents glaucomatous retinal degeneration^10^ in excitatory amino acid carrier 1 (EAAC1) knockout (KO) mice, a mouse model of normal tension glaucoma (NTG)^11,12^. Ripasudil strongly suppressed oxidative stress-induced phosphorylation of p38 mitogen-activated protein kinase (MAPK) that stimulates RGC death in EAAC1 KO mice^10,13^. These results suggest that ripasudil promotes RGC protection by stimulating both IOP-dependent and IOP-independent pathways. In addition, oral ripasudil partially prevented ONC-induced RGC death by suppressing oxidative stress via Nox1 downregulation^14^. In the present study, we examined the effects of topical ripasudil on RGC protection and axon regeneration using an ONC model.

## Results

### Ripasudil protects RGCs after optic nerve injury

**Figure 1:**
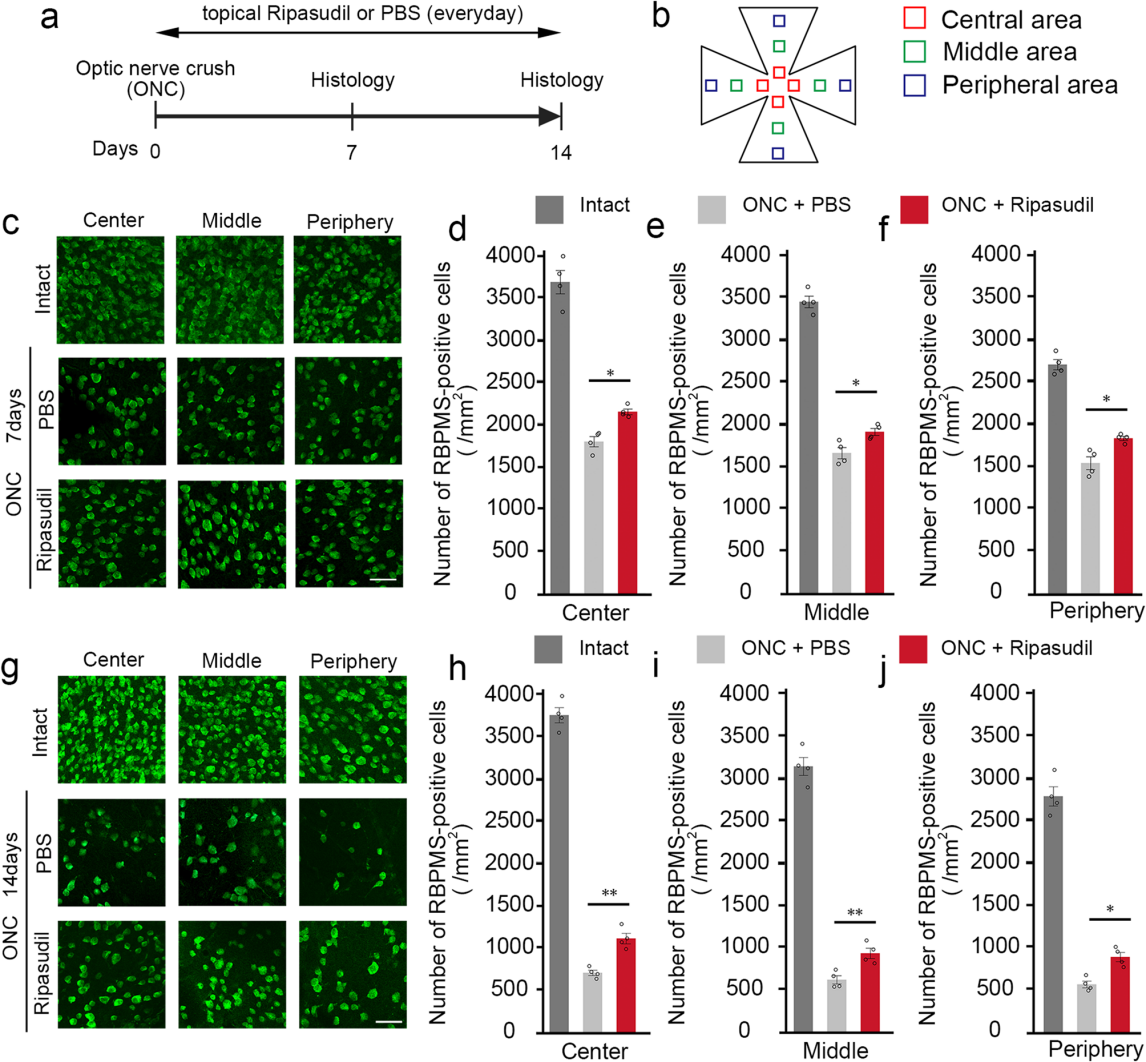
Effects of topical ripasudil on RGC degeneration following ONC. (**a**) Experimental protocol. Ripasudil (2%; 5 µl) or PBS (5 µl) was administered locally every day, and the mice were euthanized at day 7 or 14 after ONC. (**b**) Schematic illustration of the retinal areas. (**c**) Immunostaining of the RBPMS-positive RGCs in the central, middle, and peripheral areas of the retina at 7 days after ONC. Scale bar: 50 µm. (**d**–**f**) Quantification of RGCs in the central (**d**), middle (**e**), and peripheral (**f**) areas of the retina. (**g**) Immunostaining of the RBPMS-positive RGCs in the central, middle, and peripheral areas of the retina at 14 days after ONC. Scale bar: 50 µm. (**h**–**j**) Quantification of RGCs in the central (**h**), middle (**i**), and peripheral (**j**) areas of the retina. *n* = 4 per group. The data are presented as means ± SEM. *P < 0.05, **P < 0.01.

To investigate whether ripasudil protects RGCs following ONC, we topically administered daily ripasudil or phosphate-buffered saline (PBS) for 14 days (Fig. 1a). To detect RGCs, we performed immunohistochemical analysis of the flat-mounted retina with an antibody against RNA-binding protein with multiple splicing (RBPMS). We divided the areas of the retina as shown in Fig. 1b for analysis. As shown in representative pictures (Fig. 1c), ONC severely decreased the RGC density, but ripasudil partially suppressed the RGC loss in the central, middle, and peripheral areas at day 7 (Fig. 1d–f). Similar protective effects were detected in all the area at day 14 (Fig. 1g–j). These data demonstrate that ripasudil prevents RGC death all across the retina following ONC.

### Ripasudil decreases phosphorylation of p38 in RGCs after optic nerve injury

**Figure 2:**
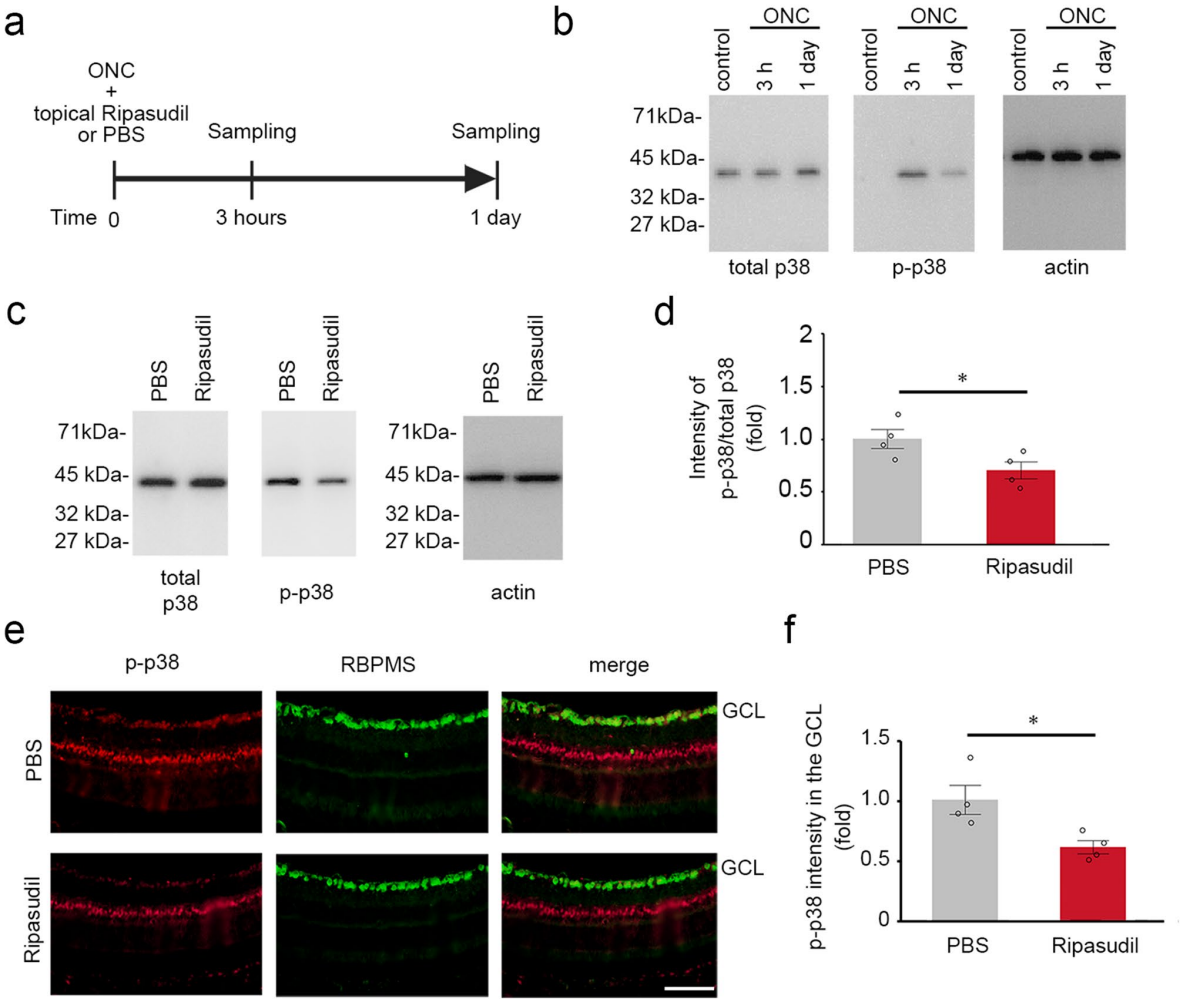
Effects of topical ripasudil on phosphorylation of p38 in the retinas following ONC. (**a**) Experimental protocol. Ripasudil (2%; 5 µl) or PBS (5 µl) was administered locally 3 min after ONC, and the mice were euthanized at 3 h and 1 day after ONC. (**b**) Immunoblot analysis of total p38, phosphorylated p38 (p-p38) and actin in the retinas at 0, 3 h and 1 day after ONC. Full length blot images are presented in Supplementary Fig. 1a. (**c**) Immunoblot analysis of total p38, p-p38 and actin in the retinas at 3 h after ONC. Full length blot images are presented in Supplementary Fig. 1b. (**d**) Quantitative analyses of (**c**). Ratio of p-p38 to total p38 in PBS-treated mice was estimated as 1.0. (**e**) Double-labeling immunohistochemistry of retinas with anti-p-p38 and anti-RBPMS antibodies. Scale bar: 50 µm. (**f**) Quantitative analyses of (**e**). The p-p38 intensity at the GCL in PBS-treated mice was estimated as 1.0. *n* = 4 per group. The data are presented as means ± SEM. *P < 0.05.

We previously reported that the topical administration of ripasudil reduces the activation of p38 in EAAC1 KO mice, which leads to suppression of glaucomatous neurodegeneration^10^. In addition, intravitreal injection of a p38 inhibitor partially suppressed RGC loss after ONC^1^. Thus, we evaluated the protein levels of total p38 and phosphorylated p38 (p-p38) in the retinas after ONC (Fig. 2a). As we previously reported^1^, p-p38 expression was increased at 3 h, but not 24 h after ONC, while total p38 expression was stable (Fig. 2b). We found that ripasudil significantly suppressed the ratio of p-p38 to total p38 compared with PBS at 3 h after ONC (Fig. 2c,d). We observed no marked changes in the protein levels of total p38 in both groups. We also prepared retinal cryo-sections at 3 h after ONC and performed immunohistochemical analysis. We found that p-p38 was mainly observed in the ganglion cell layer (GCL) and inner nuclear layer in PBS-treated mice, and p-p38 in the GCL was double-labeled with RBPMS (Fig. 2e). However, p-p38 expression in the GCL was sparse in ripasudil-treated mice (Fig. 2e). Quantitative analyses confirmed that the phosphorylation of p38 in the GCL is significantly suppressed with ripasudil treatment compared with PBS treatment (Fig. 2f).

### Ripasudil stimulates axon regeneration after optic nerve injury

**Figure 3:**
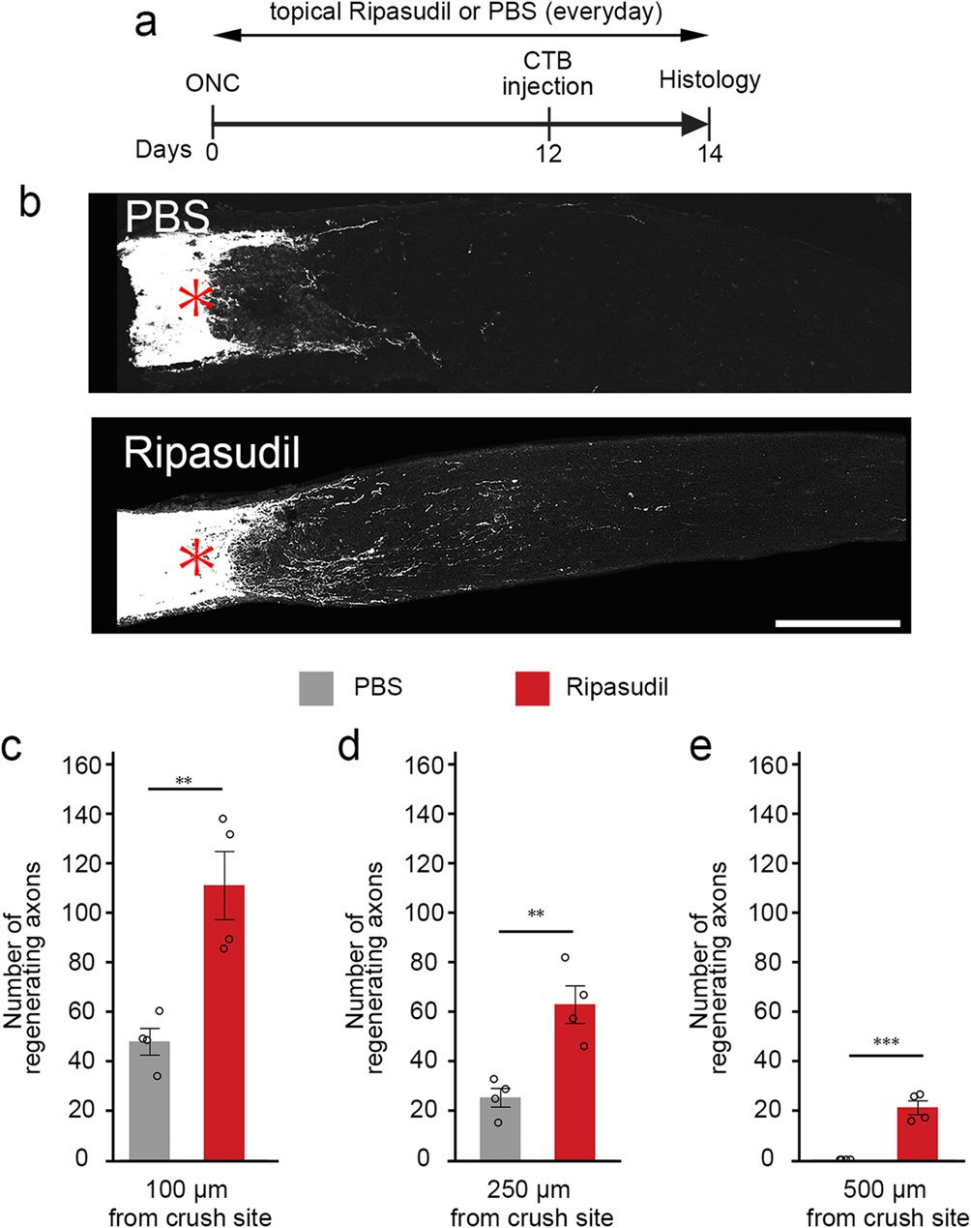
Effects of topical ripasudil on optic nerve regeneration following ONC. (**a**) Experimental protocol. Ripasudil (2%; 5 µl) or PBS (5 µl) was administered locally every day. CTB was intravitreally injected at day 12 and the mice were euthanized at day 14 after ONC. (**b**) Longitudinal sections through the optic nerve showing CTB-positive axons distal to the injury site (*) in PBS- and ripasudil-treated mice. Scale bar: 200 µm. (**c**–**e**) Quantitative analyses of regenerating axons extending 100 (**c**), 250 (**d**), and 500 (**e**) µm from the injury site. *n* = 4 per group. The data are presented as means ± SEM. **P < 0.01, ***P < 0.001.

As intravitreal injection of Y-27632 promoted regeneration of axons in rat and cat optic nerves^6,7^, we hypothesized that topical ripasudil may also have beneficial effects on optic nerve regeneration. Topical ripasudil or PBS was applied everyday after ONC and the regenerating axons of RGCs were traced by injecting cholera toxin β-subunit (CTB) into the vitreous at day 12 (d12) after ONC (Fig. 3a). Following ONC at d14, the number of regenerating axons in ripasudil-treated mice was greater than PBS-treated control mice (Fig. 3b). Quantitative analyses demonstrated that the number of regenerating axons that extended beyond 100 µm was 47 ± 10 in control and 110 ± 13 with ripasudil treatment, and beyond 250 µm was 25 ± 4 in control and 62 ± 8 with ripasudil treatment (Fig. 3c,d). In addition, 21 ± 3 axons regenerated over 500 µm in ripasudil-treated mice (Fig. 3e). These results suggest that topical ripasudil stimulates axon regeneration following ONC.

### Ripasudil suppresses phosphorylation of CRMP2 and cofilin in RGCs

**Figure 4:**
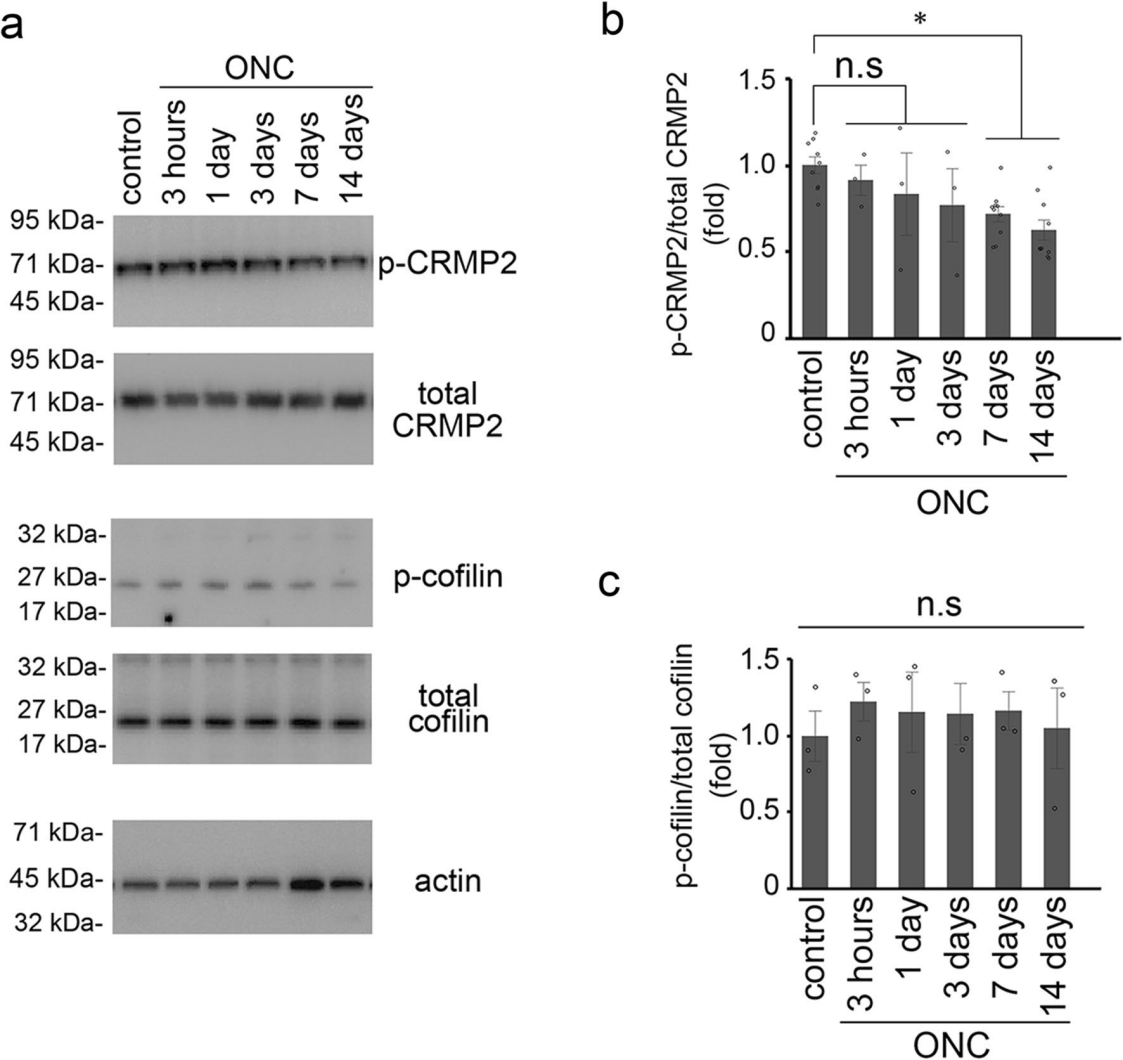
Expression of phosphorylated CRMP2 and cofilin in the retinas following ONC. (**a**) Immunoblot analysis of phosphorylated CRMP2 (p-CRMP2), total CRMP2, phosphorylated cofilin (p-cofilin), total cofilin and actin in the retina at 3 h, 1, 3, 7, and 14 days after ONC. Full length blot images are presented in Supplementary Fig. 1c and d. (**b**) Quantitative analyses of the ratio of p-CRMP2 to total CRMP2 in (**a**). Ratio of p-CRMP2 to total CRMP2 in control mice was estimated as 1.0. *n* = 9 (control), 3 (3 h, 1 and 3 days) or 10 (7 and 14 days). (**c**) Quantitative analyses of the ratio of p-cofilin to total cofilin in (**a**). Ratio of p-cofilin to total cofilin in control mice was estimated as 1.0. *n* = 3 per group. The data are presented as means ± SEM. *P < 0.05.

Downstream of the Rho/ROCK signaling pathway, phosphorylation (inactivation) of collapsin response mediator protein 2 (CRMP2), which interacts with tubulin heterodimers and facilitates microtubule assembly, inhibits neurite outgrowth^4,15^. Thus, we examined the effects of ONC on the phosphorylation of CRMP2. p-CRMP2 expression was detected in control mice without ONC, and its expression level was almost stable at 3 h, 1 and 3 days after ONC, but it seemed to be decreased at 7 and 14 days (Fig. 4a). Quantitative analyses confirmed that p-CRMP2 expression was significantly decreased at 7 and 14 days after ONC, but not at 3 h, 1 and 3 days (Fig. 4b). In addition to CRMP2, inactivation of cofilin by LIM (Lin-11, Isl-1, and Mec-3) kinase stabilizes the actin filament in the growth cone, resulting in the inhibition of neurite outgrowth^4^. We also examined the effects of ONC on total cofilin and p-cofilin expression levels using same samples and found no significant changes across all time points tested (3 h, 1, 3, 7, and 14 days after ONC; Fig. 4a,c).

**Figure 5:**
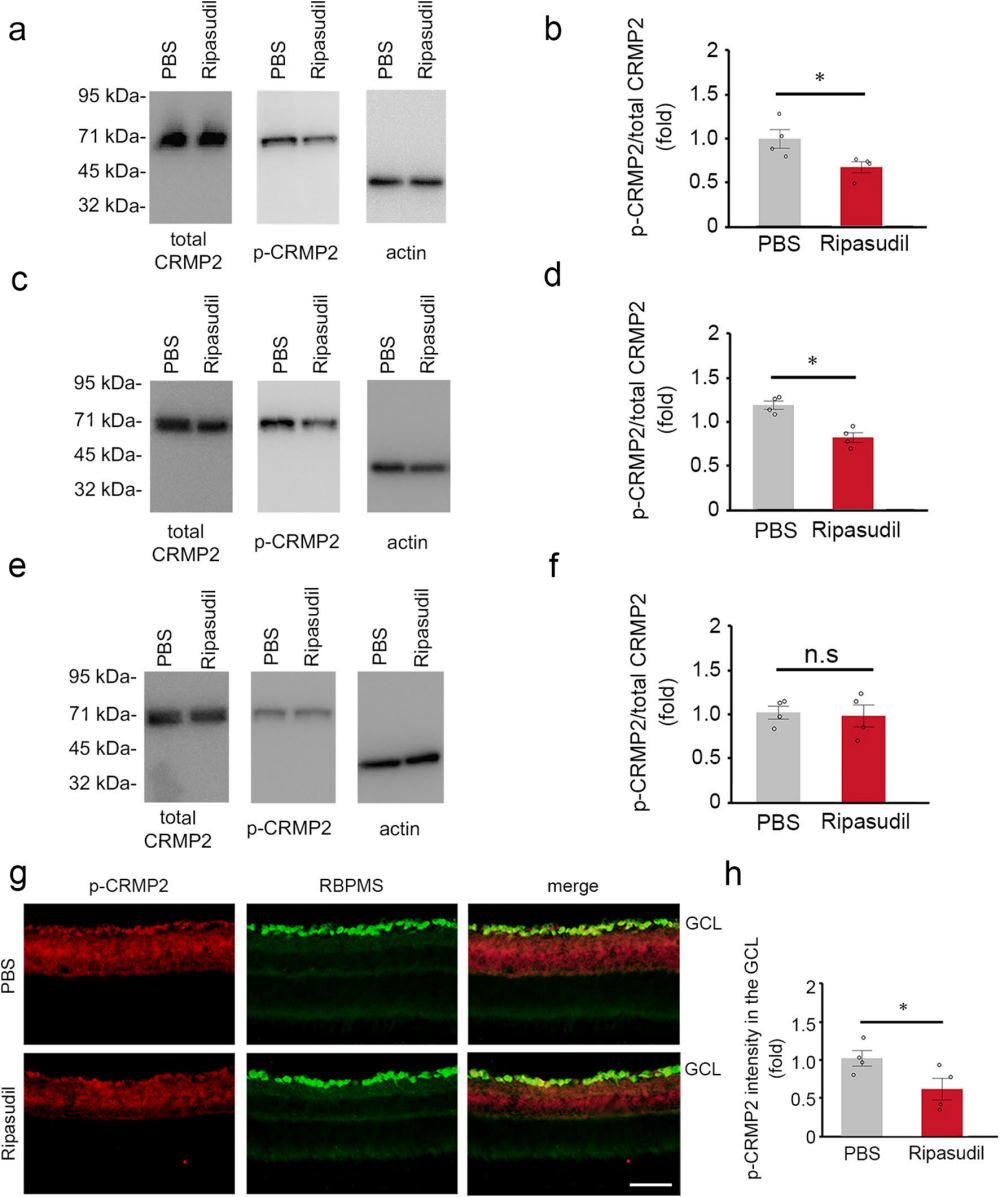
Effects of topical ripasudil on CRMP2 phosphorylation in the retinas after ONC. (**a**) Immunoblot analysis of total CRMP2 and p-CRMP2 in the retinas at 3 h after ONC. Full length blot images are presented in Supplementary Fig. 2a. (**b**) Quantitative analyses of (**a**). (**c**) Immunoblot analysis of total CRMP2 and p-CRMP2 in the retinas at 3 days after ONC. Full length blot images are presented in Supplementary Fig. 2b. (**d**) Quantitative analyses of (**c**). (**e**) Immunoblot analysis of total CRMP2 and p-CRMP2 in the retinas at 7 days after ONC. Full length blot images are presented in Supplementary Fig. 2c. (**f**) Quantitative analyses of (**e**). Ratio of p-CRMP2 to total CRMP2 in PBS-treated mice was estimated as 1.0. (**g**) Double-labeling immunohistochemistry of retinas with anti-p-CRMP2 and anti-RBPMS antibodies. Scale bar: 50 µm. (**h**) Quantitative analyses of (**g**). The p-CRMP2 intensity at the GCL in PBS-treated mice was estimated as 1.0. *n* = 4 per group. The data are presented as means ± SEM. *P < 0.05.

**Figure 6:**
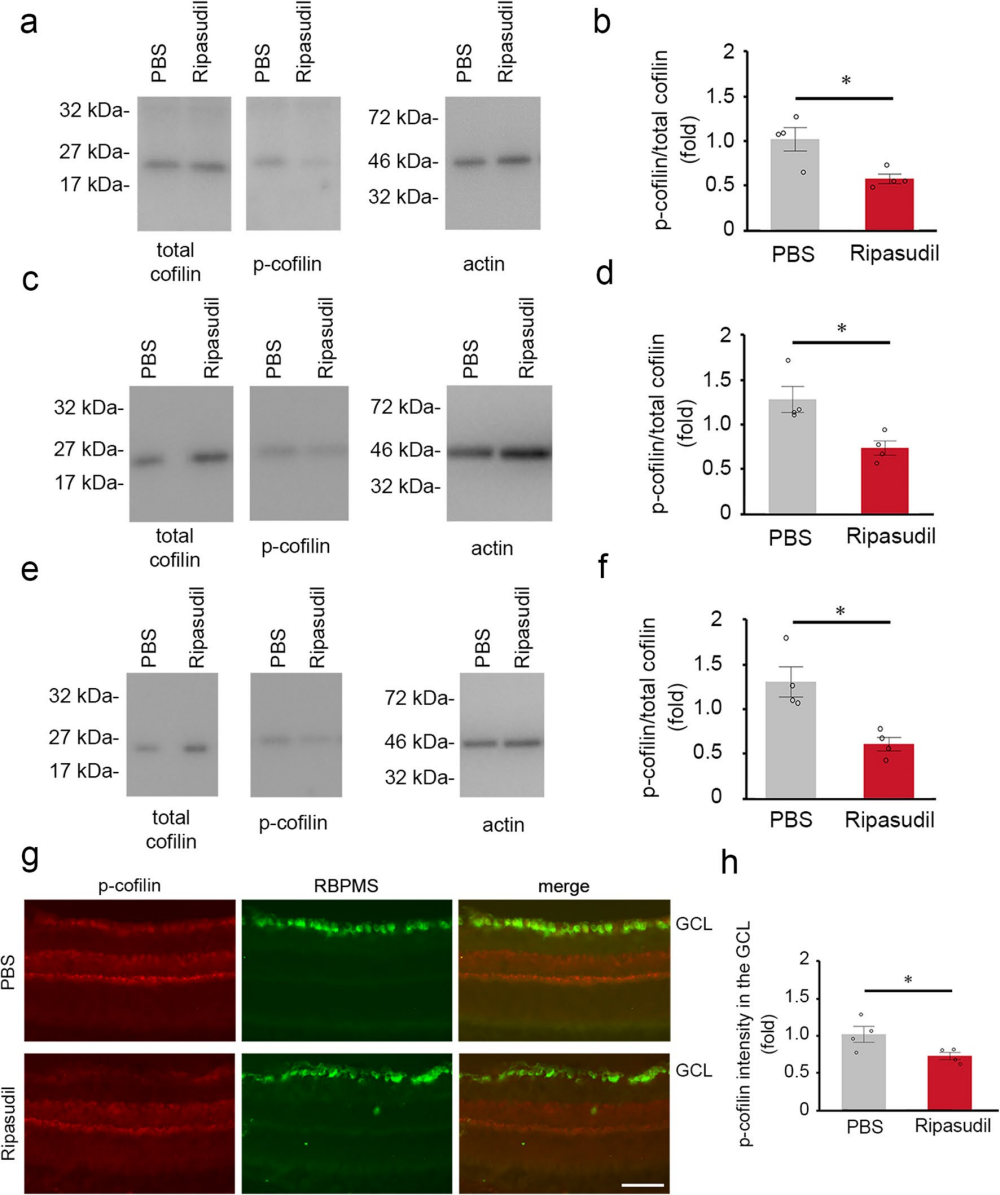
Effects of topical ripasudil on cofilin phosphorylation in the retinas after ONC. (**a**) Immunoblot analysis of total cofilin and p-cofilin in the retinas at 3 h after ONC. Full length blot images are presented in Supplementary Fig. 2d. (**b**) Quantitative analyses of (**a**). (**c**) Immunoblot analysis of total cofilin and p-cofilin in the retinas at 3 days after ONC. Full length blot images are presented in Supplementary Fig. 2e. (**d**) Quantitative analyses of (**c**). (**e**) Immunoblot analysis of total cofilin and p-cofilin in the retinas at 7 days after ONC. Full length blot images are presented in Supplementary Fig. 2f. (**f**) Quantitative analyses of (**e**). Ratio of p-cofilin to total cofilin in PBS-treated mice was estimated as 1.0. (**g**) Double-labeling immunohistochemistry of retinas with anti-p-cofilin and anti-RBPMS antibodies. Scale bar: 50 µm. (**h**) Quantitative analyses of (**g**). The p-cofilin intensity at the GCL in PBS-treated mice was estimated as 1.0. *n* = 4 per group. The data are presented as means ± SEM. *P < 0.05.

We next examined if topical ripasudil suppresses the phosphorylation of CRMP2 in the retina after ONC. For this purpose, we evaluated the protein levels of total CRMP2 and p-CRMP2 in the retinas after ONC with topical ripasudil or PBS treatment. We found that ripasudil significantly suppressed the ratio of p-CRMP2 to total CRMP2 compared with PBS at 3 h and 3 days, but not 7 days after ONC (Fig. 5a–f). We observed no marked changes in the protein levels of total CRMP2 in both groups. We also performed immunohistochemical analysis at 3 h after ONC and found that p-CRMP2 was observed in the inner retina in PBS-treated mice and double-labeled with RBPMS in the GCL (Fig. 5g). However, p-CRMP2 expression was suppressed in ripasudil-treated mice (Fig. 5g). Quantitative analyses confirmed that the phosphorylation of CRMP2 in the GCL is significantly suppressed with ripasudil treatment compared with PBS treatment (Fig. 5h). We also evaluated the protein levels of total cofilin and p-cofilin in the same retinas. We found that ripasudil significantly suppressed the ratio of p-cofilin compared with PBS at 3 h, 3 and 7 days after ONC (Fig. 6a–f). We observed no marked changes in the protein levels of total cofilin in both groups. Immunohistochemical analysis revealed that p-cofilin was observed in the inner retina in PBS-treated mice, and its expression was suppressed in ripasudil-treated mice (Fig. 6g). Quantitative analyses confirmed that the phosphorylation of cofilin in the GCL is significantly suppressed with ripasudil treatment compared with PBS treatment (Fig. 6h).

## Discussion

In this study, we showed that topical administration of ripasudil partially suppressed ONC-induced RGC death. ONC is known to stimulate the production of reactive oxygen species and activates the stress-responsive signaling pathways such as the apoptosis signal-regulating kinase 1 (ASK1)-p38 pathway, and intraocular injection of a p38 inhibitor partially prevents ONC-induced RGC death^1,16^. Our results are consistent with the previous reports that various ROCK inhibitors suppress stress-activated MAPK family members including p38^17,18^. One may think that topical administration in mice often allows the drug to reach the retina much easier than larger animals, and argue that the current results may not be meaningful in human patients. However, recent study in humans reported that topically applied ophthalmic solution may penetrate into the vitreous more than we expect, and this may be independent of the lens status^19^. We previously reported that topical application of brimonidine, an α2-adrenergic receptor agonist, exerts neuroprotective effects in EAAC1 KO mice^20^. In addition, brimonidine eye drops may promote axon growth after ONC through Erk phosphorylation^21^. Thus, some eye drops may be useful for neuroprotection in addition to lowering IOP.

In our study, the neuroprotective effect of ripasudil was modest considering the suppression rate of p-p38 in the retina. We previously reported that some RGC subtypes are highly tolerant to neurotoxicity and glaucoma-like degeneration in glaucoma model mice^22^. Subtype-specific susceptibility to RGC death is also observed after ONC^23,24^. These results suggest that some RGCs may be affected less by changes in p-p38, and that strong suppression of p-p38 by ripasudil may not directly lead to the effective RGC protection. In addition, the modest effects of ripasudil treatment on optic nerve regeneration in this study may be due to the route of administration (eye drops). Stronger effects may be achieved by intraocular injection^6,7^ or oral administration^14^ of ripasudil, but these methods may not be ideal for clinical practice. Moreover, one may consider that additional regenerating axons may be related to the extra survival RGCs, and that the effects of ripasudil on regeneration/extension of axons are questionable. However, regenerating axons were observed in the ripasudil treatment group at a site where there was hardly any regenerating axons in the control group (500 µm from the crush site), and as ripasudil affects phosphorylation of CRMP2 and cofilin, two of the molecules that are involved in regulation of axon regeneration, it is likely that topical ripasudil was able to increase the ability of axons to regenerate.

Inactivation of CRMP2 inhibits tubulin polymerization, leading to neurite outgrowth inhibition^25,26^. In this study, we demonstrated that topical ripasudil suppressed the inactivation of CRMP2 before 7 days after ONC in vivo. A recent study examined the effects of gene therapy using adeno-associated viral (AAV2) vectors encoding either brain-derived neurotrophic factor (BDNF) or a mutant, phospho-resistant CRMP2 (CRMP2T555A)^27^. Intravitreal administration of AAV2-BDNF or AAV2-CRMP2T555A enhanced the survival of RGCs and preserved the components of axonal and myelin structure after ONC. These results suggest that suppression of CRMP2 phosphorylation is effective for the protection of RGC axons. In addition, ripasudil suppressed the inactivation of cofilin, which facilitates actin dynamics. We previously reported that overexpression of DOCK3, a new member of the guanine nucleotide exchange factors, promotes actin dynamics by activating Rac1 and microtubule dynamics by inactivating CRMP2 and adenomatous polyposis coli (APC), which lead to axon regeneration^15,28,29^. Thus, stimulating downstream signaling of DOCK3 in combination with suppression of the ROCK/CRMP2 and ROCK/LIM kinase/cofilin pathways may be available for neurodegenerative diseases, such as glaucoma^4,29–31^. Further study will be required to determine the long-term effect of ripasudil on the activation of p38, CRMP2 and cofilin in ONC and other animal models of glaucoma.

## Methods

### Animals

Experiments were performed using 8- to 10-week-old C57BL/6J mice (CLEA Japan, Tokyo, Japan) in accordance with the ARVO Statement for the Use of Animals in Ophthalmic and Vision Research. Animal experiments were approved by the Institutional Animal Care and Use Committee of the Tokyo Metropolitan Institute of Medical Science (Approval number TMiMS: 19,024).

### ONC and anterograde labeling

Mice were anesthetized with 2% isoflurane before ONC. Optic nerves were exposed intraorbitally and crushed at approximately 0.5–1.0 mm from the posterior pole of the eyeball with fine surgical forceps for 5 seconds^1,2,28^. On d12 after ONC, 2 µg of Alexa 647-conjugated CTB (Invitrogen, Carlsbad, CA, USA) was injected into the vitreous body. On d14 after ONC, the animals were perfused with Zamboni’s Fixative (2% paraformaldehyde and 15% picric acid in 0.1 M phosphate buffer). The optic nerve was removed, postfixed and immersed in 30% sucrose overnight at 4 °C. The optic nerve was then embedded in an OCT compound (Sakura, Tokyo, Japan), frozen on dry ice and 14-µm serial cross-sections were prepared using a cryostat and collected on MAS-coated glass slides (Matsunami, Osaka, Japan). To estimate the total number of regenerating axons, axonal outgrowth was quantified by counting CTB-positive axons that crossed a virtual line parallel to the lesion site at 100, 250 and 500 µm distal to the lesion site.

### Drug administration

Mice received once-daily topical administration (5 µl/day) of PBS as the control or 2% ripasudil (Kowa Co. Ltd., Tokyo, Japan) solution in PBS. We selected this dose of ripasudil because 2% is the concentration that can be stably dissolved without precipitation at normal temperature, and 2% ripasudil was effective for RGC protection in EAAC1 KO mice^10^. 2% ripasudil or PBS was administered 3 min after ONC. Once the surgery was completed, the mice were assigned identification numbers randomly so that the nature of treatment (vehicle or ripasudil) was masked to evaluators of the results.

### Immunohistochemistry and morphometric studies

Mice were perfused with Zamboni’s fixative and eyes were enucleated, postfixed in Zamboni’s fixative for 2 h at 4℃. Retinas were then isolated from the eyecup, incised radially into four radial pieces^22^. For immunolabeling with an RBPMS antibody, the retinas were first incubated for 2 h in a blocking solution containing 5% horse serum and 1% Triton X-100 in PBS (pH 7.4). The retinas were then incubated in a primary antibody against RBPMS (1:1000; host, guinea-pig; ABN1376; Merck, Kenilworth, NJ, USA) at 4 °C for 2 days. After washing three times in PBS, the samples were incubated with the secondary antibody (1:1000; donkey anti-guinea pig Alexa Fluor 488; AB_2340472; Jackson Immuno Research Laboratories, West Grove, PA, USA) for 1 day. The retinal wholemounts were examined with a fluorescence microscope (BZ-X800; Keyence, Osaka, Japan). The density of immunopositive RGCs were obtained from four central (0.1 mm from the optic disc), four middle (0.8 mm from the optic disc), and four peripheral (1.5 mm from the optic disc) areas (0.04 mm^2^) of each retina^22^. RGCs were counted manually by three people, blind to the treatments. The average density of RGCs per square millimeter was calculated.

For double-labeling immunohistochemistry, mice were perfused with Zamboni’s fixative and eyes were postfixed in Zamboni’s fixative for 2 h, and then transferred into 30% sucrose overnight at 4 °C. Retinal cryostat sections of 10-µm thickness were cut through the optic nerve and examined by immunostaining using an antibody against RBPMS (1:1000), p-p38 (1:1000; 9211 s; Cell Signaling Technology, Danvers, MA, USA), p-CRMP2 (1:1000; CP2191; ECM Biosciences, Versailles, KY, USA), or p-cofilin (1:1000; 3311 s; Cell Signaling). Samples were then incubated with the appropriate secondary antibodies (for RBPMS, 1:1000; donkey anti-guinea pig Alexa Fluor 488; AB_2340472; Jackson Immuno Research; and for others, 1:1000; goat anti-rabbit Alexa Fluor 568; ab175471; Abcam, Cambridge, UK) for 1 day. The intensities of p-p38, p-CRMP2 and p-cofilin at the GCL were analyzed at 0.4 mm from the optic disc in three sections per eye using ImageJ (version2.0.0-rc-69/1.52p) (https://imagej.net/Fiji) under the same relative gain and threshold settings^32^.

### Immunoblot analyses

Immunoblotting of the whole retina was performed as previously reported^33^. Membranes were incubated with an antibody against p38 (1:1000; sc-535; Santa Cruz, Santa Cruz, CA, USA), CRMP2 (1:1000; 11,096; Immuno-Biological Laboratories, Gunma, Japan), cofilin (1:1000; 612,144; BD, Franklin Lakes, NJ, USA), p-p38 (1:1000), p-CRMP2 (1:1000), p-cofilin (1:1000) or actin (1:1000; 612,656; Becton–Dickinson, San Jose, CA, USA). The band intensities were analyzed using ImageJ (version2.0.0-rc-69/1.52p). Full-length blot images are presented in Supplementary Figs. 1 and 2.

### Statistics

Data are presented as means ± SEM. When statistical analyses were performed, the Student’s *t*-test or 1-way ANOVA followed by a Tukey’s post hoc test was used. *P* < 0.05 was regarded as statistically significant. JMP version 15.0.0 (SAS Institute Inc., Cary, NC, USA) was used for statistical analyses.

## Supplementary information

Supplementary file1.

## Data Availability

The datasets generated during and/or analysed during the current study are available from the corresponding author on reasonable request.
