# Supplementary material for: Topical ripasudil stimulates neuroprotection and axon regeneration in adult mice following optic nerve injury
Source: Sci Rep. 2020 Sep 24;10:15709. doi: 10.1038/s41598-020-72748-3 (PMC7515881; doi:10.1038/s41598-020-72748-3)

Supplementary Information for

Topical ripasudil stimulates neuroprotection and axon regeneration in adult mice following optic nerve injury

Euido Nishijima, Kazuhiko Namekata, Atsuko Kimura, Xiaoli Guo, Chikako Harada, Takahiko Noro, Tadashi Nakano and Takayuki Harada

Supplementary figure 1

Original blots for Figures 2b (**a**), 2c (**b**), 4a (**c**) and 4c (**d**).

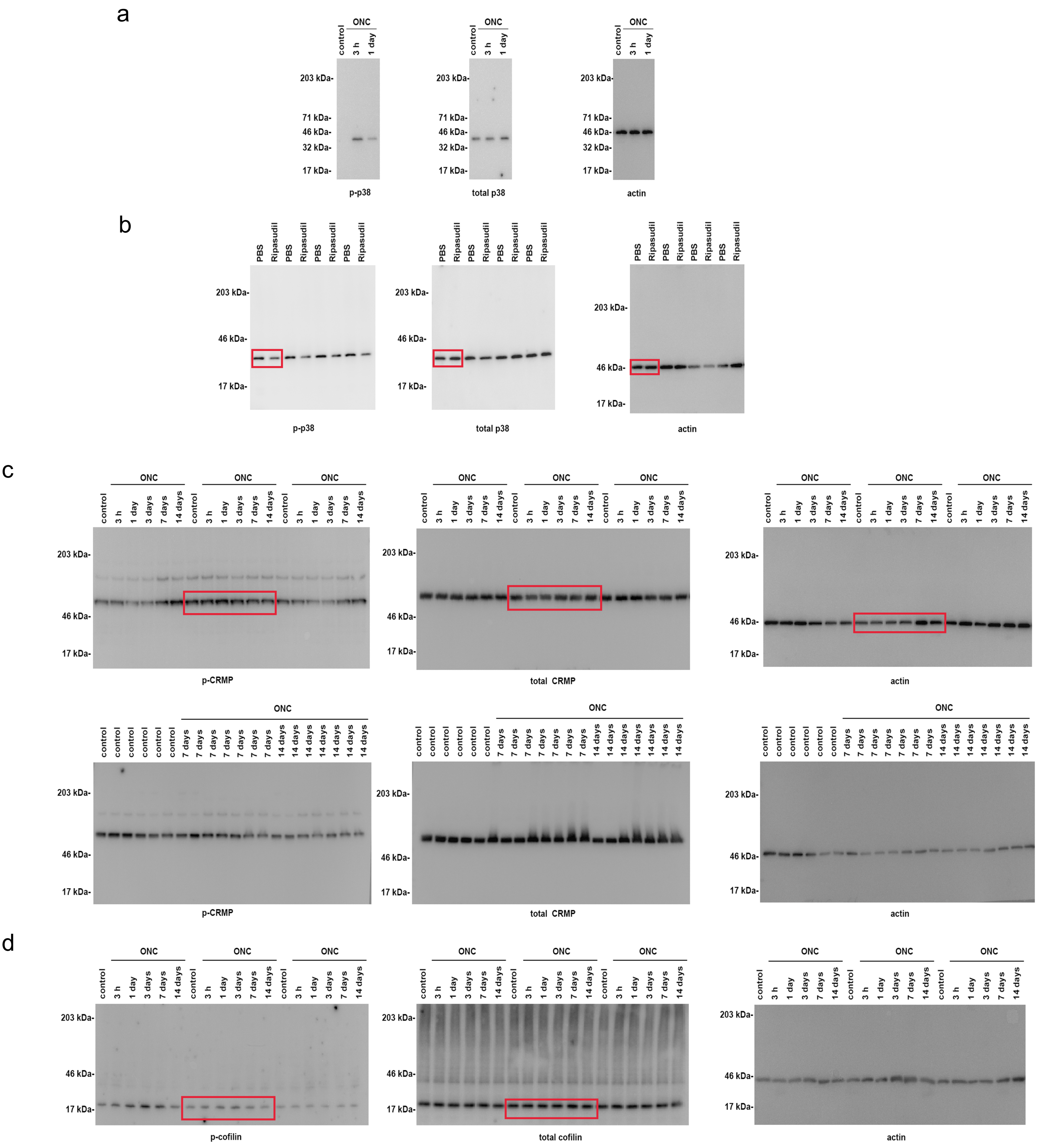

Supplementary figure 2

Original blots for Figures 5a (**a**), 5c (**b**), 5e (**c**), 6a (**d**), 6c (**e**) and 6e (**f**).

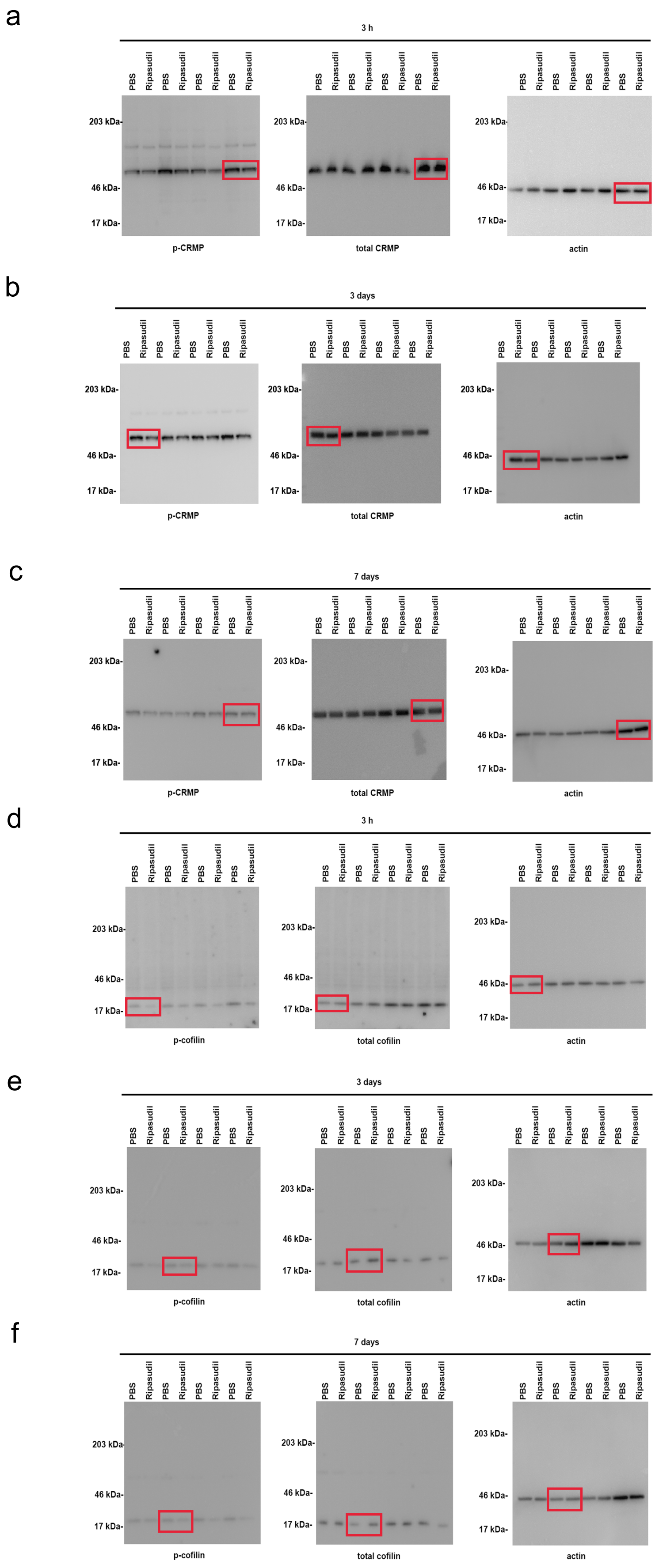

Supplement: Supplementary file 1 — Supplementary file1. [file 41598_2020_72748_MOESM1_ESM.pdf]
